# Autonomous Shepherding Behaviors of Multiple Target Steering Robots

**DOI:** 10.3390/s17122729

**Published:** 2017-11-25

**Authors:** Wonki Lee, DaeEun Kim

**Affiliations:** Biological Cybernetics Lab, School of Electrical and Electronic Engineering, Yonsei University, 50 Yonsei-ro, Seodaemun-gu, Seoul 120-749, Korea; wonkilee@yonsei.ac.kr

**Keywords:** shepherding task, nearest-neighbor interactions, multiple steering agents, collecting behavior

## Abstract

This paper presents a distributed coordination methodology for multi-robot systems, based on nearest-neighbor interactions. Among many interesting tasks that may be performed using swarm robots, we propose a biologically-inspired control law for a shepherding task, whereby a group of external agents drives another group of agents to a desired location. First, we generated sheep-like robots that act like a flock. We assume that each agent is capable of measuring the relative location and velocity to each of its neighbors within a limited sensing area. Then, we designed a control strategy for shepherd-like robots that have information regarding where to go and a steering ability to control the flock, according to the robots’ position relative to the flock. We define several independent behavior rules; each agent calculates to what extent it will move by summarizing each rule. The flocking sheep agents detect the steering agents and try to avoid them; this tendency leads to movement of the flock. Each steering agent only needs to focus on guiding the nearest flocking agent to the desired location. Without centralized coordination, multiple steering agents produce an arc formation to control the flock effectively. In addition, we propose a new rule for collecting behavior, whereby a scattered flock or multiple flocks are consolidated. From simulation results with multiple robots, we show that each robot performs actions for the shepherding behavior, and only a few steering agents are needed to control the whole flock. The results are displayed in maps that trace the paths of the flock and steering robots. Performance is evaluated via time cost and path accuracy to demonstrate the effectiveness of this approach.

## 1. Introduction

For a long time, various animal behaviors have been a source of inspiration to mankind. In many areas, the abilities of animals still surpass those of humans. By studying how animals survive in their environment, we can find useful solutions to solve dynamic problems and obtain improved performance in many applications. In this paper, we consider the flocking behavior that some creatures, such as birds, fishes, ants and sheep, use to organize themselves effectively into larger groups. It is the cohesive and aligned movement of a group of individuals in a common direction. The term flocking refers to many types of flock-like behaviors: flocks, shoals and swarms.

Over the past few years, increasing attention has been directed towards the problem of coordinated control of multiple autonomous agents. The main objective is to design coordination algorithms for multi-agent networks to achieve some global objectives using local rules. This may be one example of the distributed sensor network, and such problems have been studied from the perspectives of ecology and evolutionary biology to control theory with networks of mobile agents. Many researchers have sought to understand how a group of moving objects, such as flocks of birds, schools of fish or crowds of people, can perform collective tasks such as reaching a consensus, division of labor or moving in formation [1,2]. For a group of autonomous mobile robots, flocking can be very useful to navigate in an unknown environment or avoid collisions between robots and obstacles. If each individual has a limited sensing ability, forming a swarm could improve the sensing ability at the swarm level.

All studies of flocking of various types have been inspired by the work of Reynolds [3], which was the first simulated flocking of birds based on three simple rules: collision avoidance, centering and velocity matching. Collision avoidance is used to keep distance from other agents, and flocking centering is the characteristic of sheep agents remaining close to the flock. Velocity matching with other agents is used to organize the flock. Another model of simulated flocking behavior has been proposed [4], which consists of the flocking behaviors: homing, whereby each agent of the flock stays in its current location; velocity matching, whereby each agent attempts to move with a certain pre-defined speed; and interaction, whereby if neighboring agents are close, they move apart, and if they are too distant, they do not influence each other, otherwise they move closer together. These two models remain the basis of flocking simulations and are widely used in current implementations.

Shepherding is an interesting flocking behavior, in which one or more external agents (called steering agents) guide another group of agents (called flocking agents or sheep agents in contrast to shepherding agents). In nature, we can observe that many animals that move in groups decide their movement depending on interactions among group members. For example, other entities could be sheepdogs or predators (e.g., wolves); each sheep has a tendency to maintain its distance from the sheepdogs or predators. Generally, only a few individuals in groups have specific information, such as knowledge about the location of a food source, or a migration route from the current area to the desired area. This behavior is easily found, e.g., in sheep herding, in which the sheep react to the shepherding by moving away from the sheepdogs. One of drawbacks of the sheepdog algorithm is that it is strongly dependent on the flocking behavior of the sheep agents. In other words, if the sheep agents stop flocking, but instead disperse, the algorithm may no longer work. In addition, the sheepdog should have map information. Unless the two conditions are satisfied, the shepherding task would be hardly achievable.

Mimicking shepherding behaviors requires control rules for sheep agents and steering agents, respectively. Flocking sheep agents use the rules needed to form the flock, and a few steering agents need additional rules for steering capability based on the environmental information; these steering agents guide the group to a desired location. In this paper, we wish to implement the shepherding task including collecting behavior with multiple steering robots. The basic algorithm to control the sheep agents is similar to the flocking model proposed in [3]. The potential function defined in [5] has been used to model collision avoidance behavior [6,7,8], and in our approach, it is extended to model flocking more mathematically in a vector form. The rule for steering agents is more complicated than the sheep agents to reflect the herding behaviors. The steering agents only need information regarding the nearest agent in a group and focus on guiding the selected sheep agent to the desired location without considering any other agents in the flock. When the steering agents move toward the flock, some flocking agents detect their approach and try to move away from the steering agents. This avoidance tendency automatically moves the flock. With multiple steering agents, their interactions are also controlled independently.

In nature, it is not possible for sheepdogs to observe all sheep because the height of the vision field of sheepdogs is below the height of sheep. In addition, it is almost infeasible to simultaneously control several dogs manually. Our proposed approach reflects these realistic limitations. From simulation experiments using multiple robots, we show that the proposed method can perform the given shepherding task effectively. Only a small number of steering agents is needed to control the entire flock, and each steering agent performs its shepherding behavior independently. The steering agents trace arc paths autonomously, and even scattered flocks can be controlled via various shepherding behaviors, such as herding, patrolling and covering. The rest of the paper is organized as follows. Section 2 presents related work on the topic of flocking. Section 3 introduces the simulation environment, including the behavior of the robot. Section 4 proposes an approach for implementing shepherding behaviors. Section 5 shows the experiments and results, and Section 6 presents the conclusions and possible future work.

## 2. Related Works

In nature, flocking is a self-organizing phenomenon, as exemplified by a swarm of ants, a flock of birds and a school of fish. A large number of interacting agents is needed to show collective behaviors. Studies of flocking have attempted to understand those behaviors, which may inspire the development of distributed control and coordination of multiple mobile autonomous agents. In robotics and control theory, these problems have been studied in various ways, such as cooperative control of autonomous robots, unmanned vehicles and general multi-agent systems.

The first work was conducted by Reynolds [3], who simulated the motion of flocks of birds, using simple flocking rules. A similar model was studied in [9]. The model describes a set of agents moving with constant speed and the average movement direction of neighboring agents. If the neighborhood agents are connected, the movement direction of all agents converges to a common goal. A convergence analysis of flocking algorithms was provided by [10], and a theoretical analysis was presented in [11,12], which introduced three types of agents: α-agents, β-agents and γ-agents, and showed the similarity of flocking algorithms to Lyapunov stability analysis.

A flock of mobile robots equipped with sensing and communication devices served as mobile sensor networks [13,14], and a control law was presented in an obstacle-free environment [15]. It has also been demonstrated that a flock of agents builds networks with a dynamic topology that depends on the states of local agents [12,16,17,18]. More recently, new solutions to formation control have been studied, which were inspired by flocking social insects, due to various potential benefits such as robustness, flexibility and enhanced performance [19,20,21,22]. Some studies have attempted to use multiple robots to handle tasks or move objects cooperatively, such as pushing a box [23] or kicking a ball [24].

The topic of shepherding a flock of agents by robots has gained attention as it can have practical utility for many applications; robots may work cooperatively to herd a group of animals or robots [8,25,26,27,28,29,30,31,32]. A sheepdog robot has been tested to gather a flock of biological ducks and guide them safely to a desired position [26]. This was the first study to control an animal’s behavior with the shepherding idea. A potential-field model was applied to the shepherding task both in simulation and in the the real world. A herding method using a single shepherd-robot has been suggested [27,29]. A single shepherd could control the flock by adaptively positioning itself. However, it has also been shown that a single shepherd cannot adequately control a large flock or flocks whose behavior is hardly manageable. It has been explored that multiple shepherds can be used to control large flocks better [33]. They show that multiple shepherding agents can work cooperatively to effectively control another group of agents based on the impact from the shepherds and obstacles in the environment. Recently, it has been shown that multiple shepherding robots drive a herd of non-cooperative sheep agents to a goal position [34]. They provide a control law for shepherding robots using a dynamic robot model. Applications of robotic interaction (small vehicles) as one chasing another (prey-predator) can be found in [35]. Recently, there was a two-person cooperative virtual shepherding task in the aspect of human-robot interaction [32,36]. Their work examined multi-agent shepherding task, where pairs of individuals herd virtual sheep in the virtual game field. They showed that many pairs of participants spontaneously produce an effective behavior mode.

In real applications of shepherding using robots, it is commonly assumed that the steering agents receive the position information of members. All individuals are localized with the help of centralized coordination. The localization information of agents is obtained using range sensors [25], overhead camera [26], laser sensors [37,38] or wireless motion-tracking sensors [32,36]. Many navigation systems have a target, which a mobile agent is supposed to reach with various sensor readings [37,38], whereas in the shepherding task, only steering agents have target information and make an effort to guide sheep agents. Then, the observers or steering agents produce appropriate actions to control the sheep robots to form a flock or move the group of sheep robots towards the target position.

When there are multiple shepherding robots, they generally produce two types of formations, namely a line formation or an arc formation using one of the three approach locomotions: vector projection, greedy distance-minimization and global distance-minimization [33]. In these approaches, the shepherding robots share all the information about the environment; however, this is not feasible in real-world situations. In our work, each steering agent only guides the sheep agents in view in a flock, and the relative positions of steering agents are also obtained via repulsion among steering agents within a sensing area.

For autonomous shepherding behaviors, a robot should observe the surrounding environment through its own sensors. The robot needs special sensors for detecting other robots and avoiding obstacles [39]. Furthermore, localization information can be shared by other robots with communication devices. Recently, many sensing techniques for communication and detection have been developed. Several wireless technologies have been proposed to achieve reliable communication [40,41] and applied to robots applications [42,43,44,45]. In the smart transportation system, detection and ranging sensors can allow robust detection for a target and measurement of their speed, distance and motion direction [46,47,48]. In this paper, we assume that the physical layer of the sensing/communication part of the multi-agent system is well developed, and we concentrate on the algorithm of the multi-agent system especially for the shepherding task.

## 3. Description of Shepherding Task

In the real world, a sheepdog can perform many tasks. Herding, patrolling, covering and collecting are common types of shepherding behaviors [33]. Herding is the task in which the steering agents move the flock to the desired area, such as the entrance to a food source, without any dropout. After completing herding, steering agents can control the flock so that it remains in its current location, by patrolling. To keep the flock within a specific area, a rule that encourages them to stay within approximate boundaries is needed. Patrolling prevents an individual agent from escaping from the flock. Covering is the task in which the shepherd moves the flock as one unit to a different field. In the current work, the covering task consists of repeating the herding task. Shepherds move a flock of sheep as one unit to eat grass in another field. Collecting is needed for a scattered flock or multiple flocks. When there exists more than one flock or the flock is scattered, steering robots need to merge the scattered flocks.

Our study investigates the ability of the shepherding agents (steering agents) to control a flocking swarm of robots in an obstacle-free environment. The main task for the steering agents is to guide the swarm to goal areas. Steering agents guide the closest flocking agents at any given time, and the effects spread to all members of the flock that is being moved to the desired area.

### 3.1. Environment

**Figure 1:**
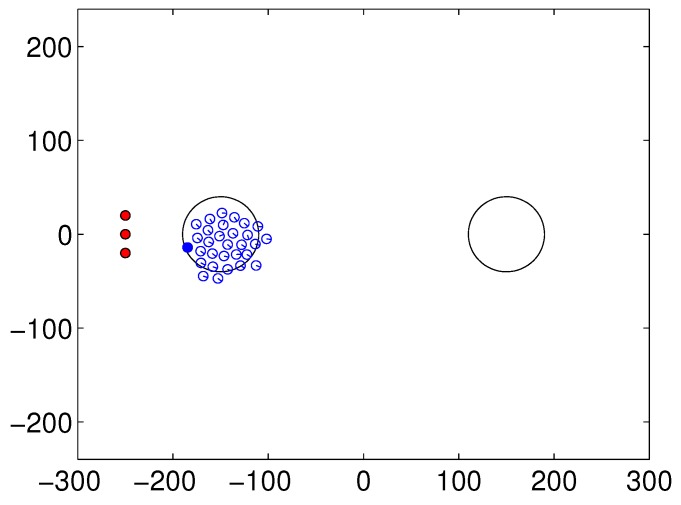
Snapshot of multi-robots for shepherding at the initial state (red-colored robots: steering robots); the left circle indicates the starting point and the right circle the destination area.

A snapshot of the experimental environment for shepherding behaviors is shown in Figure 1. We used a rectangular (600 × 500) cm obstacle-free arena. The origin (0,0) is positioned in the center of the field. The two large circles with a radius of 40 cm are located at (−150,0) cm and (150,0) cm, and there are areas to where the flock should be directed. The left-hand circle is the starting position of the flock, and the right-hand circle is the destination of the flock. The goal of moving the flock to the destination should be attained by a series of actions of the steering robots. The sheep robots are initially located randomly in the left circle with random heading direction. Steering agents are located 100 cm away to the left-hand side, and they are supposed to guide the sheep robots to the desired area. The herding behavior is complete when the center of the flock arrives in the right-hand circle. In the covering behavior, after the flock arrives at the desired area, the destination is changed to the left-hand circle.

The blue-outlined empty circles represent the sheep agents that form the flock, and the red filled circles located on the left-hand side of the flock indicate the steering agents. The steering agent chooses its target agent to control directly, the nearest agent in view among the flocking sheep agents, which is represented by the blue-colored disc in the flock. Only one agent is selected in this figure. Each steering agent selects its own target sheep agent, and the target agent may be shared by multiple steering agents.

The size of robot is 10 cm in diameter. Each robot is equipped with sensors that can measure the relative location and velocity to each of its neighbors within a limited sensing area. We assume that each agent uses the laser sensor to read the distance of other robots from the agent. The sensing range is set to 20 cm. Each steering robot can be monitored by a top view camera, and its current position relative to the goal area can be measured. To reflect real applications using real robots, noise variables, δx and δy, are added to the coordinates *x* and *y* of each sensed robot, and the position is updated each time the robot moves. When updating the current position information, a uniformly distributed value from −1.0 to 1.0 cm with the maximum ±10% error rate is added to *x* and *y*. Similarly, an error value is added to the sensed robot’s heading. The absolute values of both δx and δy are not allowed to exceed the maximum movement speed of a robot, namely 2.5 cm/s for steering agents and 1 cm/s for sheep agents, respectively. The positions of all the robots are updated synchronously.

### 3.2. Robot Behaviors

We need to define the behaviors of the two robot groups, that is the flocking sheep robots and steering (shepherding) robots. The sheep agents in the flock move based on three kinds of behavior rules to form and maintain a flock. Forming a flock for controlled group is a basic requirement to achieve the shepherding task. The information needed to form a flock are the distance to other agents within the sensing area, the distance to the center of the neighboring agents and the distance to the steering agents and obstacles. The first rule is that if a pair of agents is very close, they try to minimize their separation. Second, if two agents are minimally separated, they tend to follow the other agents. Each agent tries to move toward the center of the neighboring agents and matches velocity with them. Third, they avoid other things that are not members of the flock. This includes not only obstacles and the barrier around the arena, but also steering robots. The avoidance tendency from the steering robots allows the possibility of shepherding. For example, the sheep agents move in the opposite direction of the sheepdog robots and should also avoid the obstacles.

Steering robots detect the nearest sheep robots in a flock and approach the flock to perform four behaviors: herding, patrolling, covering and collecting. The overall behavior algorithm of the steering agents for shepherding task is summarized in Algorithm 1. After completing herding behaviors, that is after the flock arrives at the desired location, if the destination is changed, it becomes a covering behavior; or if not, it is a patrolling behavior. It depends on the change of destination.

**Algorithm 1** Shepherding task
**while** running **do** **if** there is a scattered flock **then**  **if** there are other steering robots for the current herding flock **then**   **if** there are no steering robot herding a scattered flock **then**    **if** the robot is closest to a scatted flock **then**     collecting**    else**     patrolling    **end if** **   else**    herding the scattered flock to the main flock     **end if**   **else**   herding the scattered flock into the main flock    **end if** ** else**  **if** flock arrives at destination **then**   **if** covering is needed **then**    change the destination  **   else**    patrolling      **end if** **  else**   herding the main flock    **end if**  **end if** **end while**


If there is a scattered flock, the separated groups must be collected to form a single flock. If the number of separated group is larger than the number of shepherd robots, the robot closest to the current herding flock moves around that flock to patrol and thereby maintain the current position of the flock while waiting for the other robots to perform collecting behavior. Simultaneously, the other robots arbitrarily select a scattered flock and herd it toward the original flock. When the number of steering robots is greater than the number of sub-groups, the steering robots controlling the main flock herd it to the desired destination, while the other steering robot group continuously collects the divided groups. If there is only one steering robot, the collecting behavior is also skipped. This behavior is based on the assumption that the flock is maintained well after the flock is merged. Otherwise, it may need an indefinite amount of time. Since the information about the relative location between steering robots and the flock can be inferred, allocating steering robots to each sub-group occurs adaptively, and the scatted flock can be merged. The detailed task plan is given in Algorithm 1.

To see the behavior of real robots, realistic simulations with MATLAB programming are implemented based on the model of small-sized two-wheel robots (the two-wheeled robot model follows the ActiveMedia Pioneer 3DX mobile robot, Adept Mobile robots, Amherst, NH, USA). The sensor readings or motor actions for movement are involved with random noisy signals as observed in the real robotic system.

## 4. Proposed Method

### 4.1. Modeling

We consider a group of *N* agents. Each agent *i* has the information $q_{i}$ and $p_{i}=\dot{q_{i}}$, for i=1,…,N. where $q_{i}=\left(x_{i},y_{i}\right)$ is the location of agent *i*, and its velocity is $p_{i}=\dot{q_{i}}$. Then, the speed of an agent at the next time step is determined as follows:

(1) $$p_{i}\left(n+1\right)=p_{i}\left(n\right)+u_{i}$$

where *n* is the time step and $u_{i}$ is the input to change the robot movement vector. Then, the goal is to design a control input, $u_{i}$, such that the group of mobile robots is moved to the desired area by the steering agents.

The individual agent should utilize the information regarding its neighboring agents depending on its own task. Then, the control input applied to each agent consists of four components:

(2) $$u_{i}=u_{m}+u_{o}+u_{t}+u_{g}$$

where $u_{m}$ represents the control input from homogeneous agents, $u_{o}$ refers to the input from obstacles or the barrier surrounding the arena and $u_{t}$ to that from heterogeneous agents. Furthermore, $u_{g}$ is the control input from map information, such as the home position, and this can be applied only for steering agents. To achieve herding to the desired location and covering, this parameter is needed to generate a tendency to move the flock towards a particular destination. Accordingly, we design the behavior rules needed for the sheep agents and steering agents in the following sections.

### 4.2. Control Algorithm for Flocking Sheep Agents

**Figure 2:**
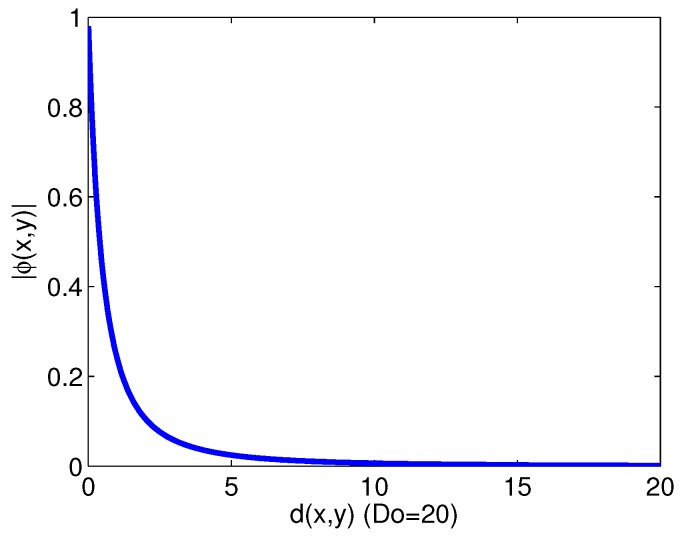
Magnitude of the function $\phi (q,N_{m})$ with distance from a neighbor agent.

For sheep agents, each individual’s position is updated based on the sum of the three rules defined in Section 3.2 and is defined as follows:

(3) $$\begin{array}{cc} u_{m} & =u_{m1}+u_{m2}+u_{m3}\\ & =K_{m1}\phi \left(q_{i},N_{m}\right)+K_{m2}\left(\frac{1}{n_{m}}\sum_{j\in N_{m}}^{}q_{j}-q_{i}\right)+\frac{K_{m3}}{n_{m}}\sum_{j\in N_{m}}^{}p_{j} \end{array}$$

where $N_{m}$ is a set of agents that can be communicated with or detected within the sensor range among the homogeneous agents, that is among group members, and $n_{m}$ is the number of agents in the set $N_{m}$. $K_{m1}$, $K_{m2}$ and $K_{m3}$ are scaling parameters; $K_{m1}$ is related to keeping a distance from other agents; $K_{m2}$ is used to remain close to the flock; and $K_{m3}$ is for velocity matching among sheep agents. The function $\phi (q_{i},N_{m})$ is defined as:

(4) $$\phi \left(q_{i},N_{m}\right)=\left\{\begin{array}{cc} {\left(\frac{1}{d(q_{i},\;\sum_{j\in N_{m}}^{}\left(q_{i}-q_{j}\right)/n_{m})}-\frac{1}{D_{o}}\right)}^{2}\frac{\sum_{j\in N_{m}}^{}\left(q_{i}-q_{j}\right)}{∥\sum_{j\in N_{m}}^{}\left(q_{i}-q_{j}\right)∥}, & \text{if }d\left(q_{i},q\right)\leq D_{o}\\ 0, & \text{if }d\left(q_{i},q\right)>D_{o} \end{array}\right.$$

where $d(q_{i},q)$ is the Euclidean distance between $q_{i}$ and *q*, *q* is the averaged vector of the neighboring agents within the detecting range and $D_{o}$ is the cutoff distance with which the collision-avoidance can be triggered in the potential field. The above term corresponds to a gradient of the potential function defined in [5], which is inversely proportional to the distance. The normalized vector sum in the product represents the unit vector to define the moving direction. The function $\phi (q_{i},N_{m})$ guides the direction away from neighbor agents, as well as calculates the potential level from a neighbor agent as shown in Figure 2.

The first term, $u_{m1}$, is that each individual with position vector $q_{i}$ and direction vector $p_{i}$ attempts to maintain a minimum distance between itself and other agents. Avoidance is given the highest priority. If no consideration is given to collision avoidance, the individual will tend to become attracted toward the center of neighbors and aligned within a local sensing range using two terms, $u_{m2}$ and $u_{m3}$. The second term is simply the average position of all the other agents within the detecting range, not including the agent itself. After calculating the center, we need to determine how to move the robot toward it, controlled by $K_{q}$. This is achieved by subtracting a vector $q_{i}$ from the averaged location of other agents. The third term indicates that each robot finds its neighbor within the detecting range and updates its velocity to match that of its neighbors.

Each agent needs to be away from any obstacle within the sensing range, and $u_{o}$ in Equation (2) can be described as:

(5) $$\begin{array}{c} u_{o}=K_{o}\phi \left(q_{i},N_{o}\right) \end{array}$$

where $N_{o}$ is a set of obstacles and $K_{o}$ is a scaling parameter.

Based on four elements, $u_{m1}$, $u_{m2}$, $u_{m3}$ and $u_{o}$, maintaining a flock is possible. However, for herding and other behaviors, such as covering and patrolling, an additional behavior rule, $u_{t}$, in Equation (2) that describes its tendency to avoid a steering robot is needed. The steering agents have relative dominance over the flock. Therefore, the sheep agents in the flock try to avoid these steering agents. This tendency can lead to the movement of the flock. If the steering agents can travel anywhere in the map, they can herd the flock to wherever is needed. For the sheep agents, this rule has the same effect as obstacles, and the vector $u_{t}$ can be defined as:

(6) $$\begin{array}{c} u_{t}=K_{t}\phi \left(q_{i},N_{t}\right) \end{array}$$

where $N_{t}$ is a set of steering agents within the detection range of a sheep agent *i*, $K_{t}$ is a scaling parameter and $\phi (q_{i},N_{t})$ is a collective potential function. Then, the overall control algorithm for sheep robots is defined as:

(7) $$\begin{array}{cc} u_{i} & =u_{m}+u_{o}+u_{t}\\ & =u_{m1}+u_{m2}+u_{m3}+u_{o}+u_{t}\\ & =K_{m1}\phi \left(q_{i},N_{m}\right)+K_{m2}\left(\frac{1}{n_{m}}\sum_{j\in N_{m}}^{}q_{j}-q_{i}\right)+\frac{K_{m3}}{n_{m}}\sum_{j\in N_{m}}^{}p_{j}+K_{o}\phi \left(q_{i},N_{o}\right)+K_{t}\phi \left(q_{i},N_{t}\right) \end{array}$$

where $K_{m1}$, $K_{m2}$, $K_{m3}$ are scaling parameters among sheep agents and $K_{o}$, $K_{t}$ are parameters between a sheep agent and the environment or steering agents, respectively. $N_{m}$ is a set of sheep agents, and $n_{m}$ is the number of elements for the set $N_{m}$. Furthermore, $q_{j}$ is the location of agent *j* and $p_{j}$ is its velocity.

The above control algorithm is the sum of vector operations depending on various factors. Each term acts independently; thus, each agent calculates how much it will move according to each rule, which provides the corresponding velocity vector. Then, those vectors are added to the robot’s current velocity to update its new movement velocity. The priority of the terms in the above control follows in sequence avoidance from obstacles, avoidance from steering robots and flocking behavior of sheep agents. This is reflected in the choice of the scaling parameters. We find it necessary to limit the magnitude of the robots’ velocities. This prevents the robots from moving too quickly; real animals or robots have limitations on how quickly they can move. To limit the speed, the unit vector is calculated by dividing the vector by its magnitude. If the magnitude exceeds the limit, the maximum movement speed is multiplied by this unit vector. Then, the vector has the same direction as the original velocity vector, but with the magnitude of the maximum movement speed.

### 4.3. Control Algorithm for Steering Agents

To control the steering agents, similar to the sheep agents, terms based on collision avoidance are commonly used. Collision avoidance of robots and obstacles is needed to control the steering agents. In particular, collision avoidance between sheep robots and steering agents is essential. When the steering agents approach or move around the flock, they must not pass through the flock, as they could disperse it. In addition, collisions among steering robots should be avoided. Then, the control algorithm for collision avoidance of steering agents is given as follows:

(8) $$\begin{array}{c} u_{m}+u_{o}+u_{t}=K_{m}^{\prime }\phi \left(q_{i},N_{m}\right)+K_{o}^{\prime }\phi \left(q_{i},N_{o}\right)+K_{t}^{\prime }\phi \left(q_{i},N_{t}\right) \end{array}$$

where $K_{m}^{\prime }$ is a scaling parameter for a group of steering agents, $K_{o}^{\prime }$ is the parameter between steering agents and obstacles and $K_{t}^{\prime }$ is the parameter between steering agents and sheep agents. For herding a flock to the destination, an additional term, $u_{g}$, is defined as follows:

(9) $$u_{g}=K_{g}^{\prime }\frac{\left(q_{t}-q_{i}\right)-\left(q_{g}-q_{t}\right)}{∥\left(q_{t}-q_{i}\right)-\left(q_{g}-q_{t}\right)∥}$$

where $K_{g}^{\prime }$ is a scaling parameter, $q_{t}$ is the location of the nearest agent and $q_{g}$ represents the goal position.

**Figure 3:**
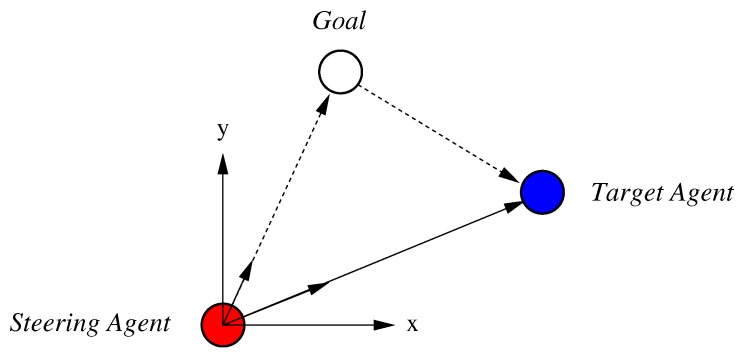
Relative position of agents and the goal position; the steering agent should move to the opposite side of the destination area.

In our method, the shepherding agents only move to the nearby sheep agents and herd the selected one in the goal direction. To herd the flock to the goal area, the steering agent should be positioned behind the flock and then move the flock to the destination. For this movement, the steering agent moves toward the nearby agent using Equation (9). Due to the collision avoidance tendency with the flock in Equation (7), it moves around the flock and positions itself autonomously such that a flock of sheep agents is between the steering agent and the goal area. As shown in Figure 3, to herd the flock, the steering agent moves to the opposite side of the destination area and pushes the flock.

Similar to the behavior rules for the sheep agents, all vector terms are summed to give the instantaneous motion vector for the steering agents. Once the sheep agents detect the approach of a steering agent, they try to avoid the steering agent and increase their distance from it. These behaviors make the flock move towards the desired goal. Then, the overall control algorithm for a steering agent can be defined as follows:

(10) $$\begin{array}{cc} u_{i} & =u_{m}+u_{o}+u_{t}+u_{g}\\ & =K_{m}^{\prime }\phi \left(q_{i},N_{m}\right)+K_{o}^{\prime }\phi \left(q_{i},N_{o}\right)+K_{t}^{\prime }\phi \left(q_{i},N_{t}\right)+K_{g}^{\prime }\frac{\left(q_{t}-q_{i}\right)-\left(q_{g}-q_{t}\right)}{∥\left(q_{t}-q_{i}\right)-\left(q_{g}-q_{t}\right)∥} \end{array}$$

where $K_{m}^{\prime }$, $K_{o}^{\prime }$, $K_{t}^{\prime }$ and $K_{g}^{\prime }$ are scaling parameters. $N_{m}$, $N_{t}$ and $N_{o}$ are a set of steering agents, a set of sheep agents and a set of obstacles within the sensing range, respectively. Furthermore, $q_{i}$ is the location of agent *i*; $q_{t}$ is the location of the nearest sheep agent in a flock; and $q_{g}$ is the location of destination. Furthermore, $\phi (q_{i},N_{m})$ is a function shown in Equation (4), but calculated with distance from steering agents.

For the herding behavior, the destination is one of circles in the arena, and for patrolling, the destination $q_{g}$ is changed to the current location of the flock. If robots are performing collecting behavior, the destination is set to the nearby flock. Table 1 shows the description of the pre-defined parameters; coordination parameters, control inputs, scaling parameters and a set of objects, and explains behaviors related to each scaling parameter to understand a potential result caused by the parameters.

## 5. Simulation Results

To analyze the algorithm, we ran a simulation experiment in which the shepherding task was executed with various parameter settings, such as the number of steering agents and the size of the flock. Generally, performance can be quantified as the normalized angular deviation of group direction around the preferred direction, the number of agents that arrive at the desired area and the time taken to perform the task. Here, we visualize the path of the center of the swarm and the path of the robot dogs. In addition, as we change the number of steering agents and the number of sheep robots, we evaluate performance as the time taken for the center of the flock to reach the desired area; this is directly proportional to the accuracy of flocking control. In the simulation, we assume that robots can distinguish steering robots and sheep robots with the help of a the beacon or LED light in a robot. Using visual information, the steering robots can detect if a flock is separated from a distance sensing range, and they can perform collecting behavior depending on the situation.

The control algorithm depends on several variables: scaling parameters, the range of perception, the size of flock and parameters of the noise distributions. The control algorithm with Equation (7) can derive different behaviors of sheep agents depending on the parameters. Sometimes, a flock of sheep agents is hardly maintained, and it is divided into several subgroups when the consolidation interaction between members in the homogeneous group is relatively weak. Initially, we set up parameters for flocking sheep agents. The coefficient parameters are set to $K_{m1}$ = 200, $K_{m2}$ = 1, $K_{m3}$ = 1, $K_{o}=1000$ and $K_{t}=500$. After confirming that the flock is maintained well, $K_{m}^{\prime }=800$, $K_{0}^{\prime }=1000$, $K_{t}^{\prime }=5000$ and $K_{g}^{\prime }=1$ are selected for the steering robots.

### 5.1. Results of Herding, Patrolling and Covering Behaviors

**Figure 4:**
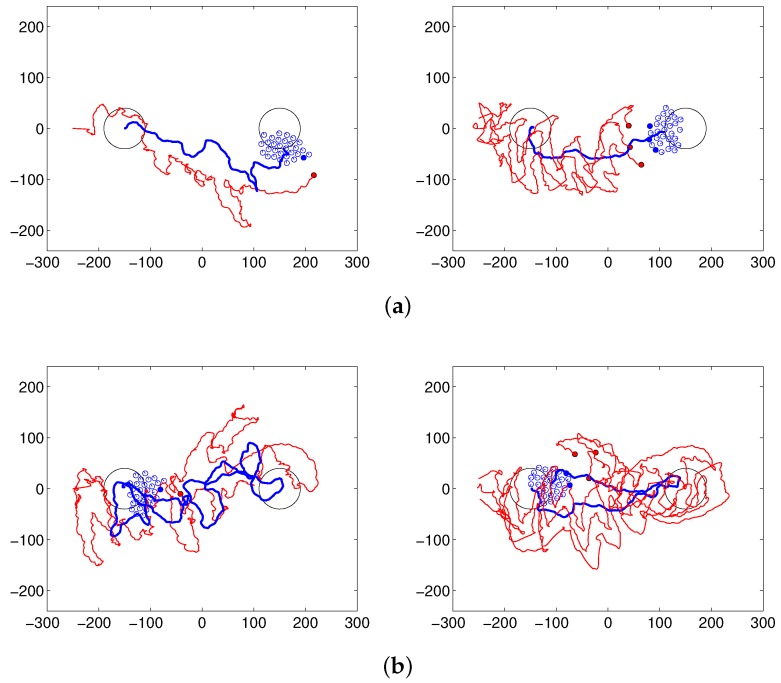

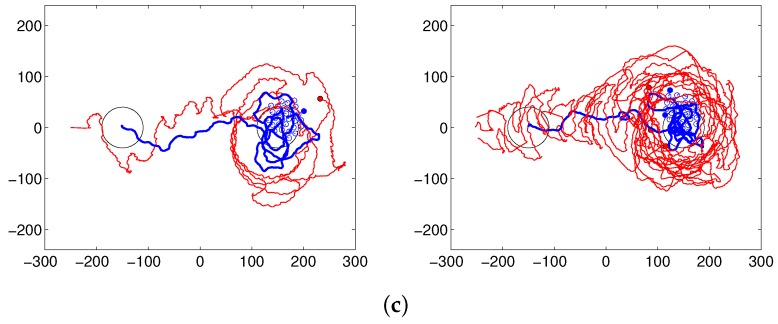
Results of (**a**) herding, (**b**) patrolling and (**c**) covering behaviors with (left panels) one and (right panels) three steering robots.

Steering robots have the ability to maintain the flock and drive it to the desired location. They directly approach a flock, repeatedly select a close agent and drive that agent to the goal. After starting from the initial position, each steering robot performs the task until the center of the flock arrives at the desired area. Figure 4 shows the trace of the center of a flock composed of 30 following robots with one (left) or three (right) steering robots. The paths of the steering robots are marked in red, and those of the center of the flock are shown in blue. Figure 4a shows the result of the herding task, and Figure 4b shows the result of the covering task, i.e., changing the destination after arriving at the initial destination. In Figure 4c, after herding the flock to the desired area, the robot performs the patrolling task to maintain the current flock position and prevent escape of the flock to other areas. The panels on the left side show the results of only one steering robot, and those on the right side show the results of three steering robots. There is less variation in the flock path for three steering robots versus one.

**Figure 5:**
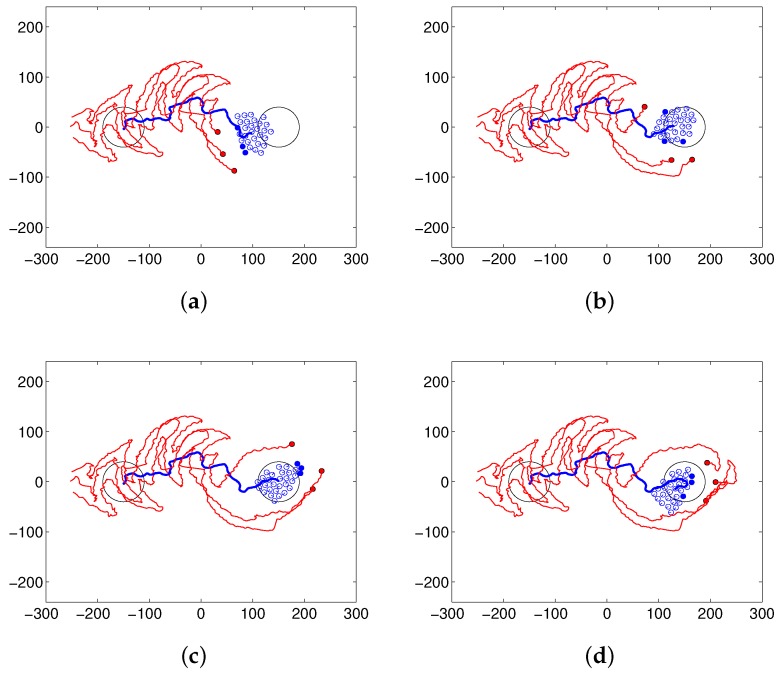
Snapshot of the simulation in which the steering robots herd the flock and perform the covering behavior without the scattered flock: (**a**) herding, (**b**,**c**) moving to the opposite side of the flock and (**d**) performing covering behavior by herding the flock to a new destination.

After reaching the goal position, steering agents move around the flock to maintain the flock and prevent the escape of the sheep agents. In so doing, the center of the flock remains in the desired area. In the covering task, the flock can be directed through predetermined points. The flock is moved to another area after arriving at the initial destination. There are many arcs that represent the movements of the steering robots. If the robots meet the flock, they trace arc paths autonomously; these paths are not enforced manually. These arcs automatically compensate for the angle of the herding. Therefore, the robots can successfully complete herding tasks. Figure 5 shows a snapshot of the simulation when the steering robots move to the opposite side of the flock after arriving at the desired area, to change the movement direction of flock and perform the covering behavior. In this case, there are three steering robots and thirty sheep robots. Figure 5a shows the arc paths that occur while the shepherding agents are herding, and Figure 5b,c shows that steering robots move around the flock and then move behind the sheep flock to guide it to a new destination, as shown in Figure 5d.

### 5.2. Results of Collecting Behavior

**Figure 6:**
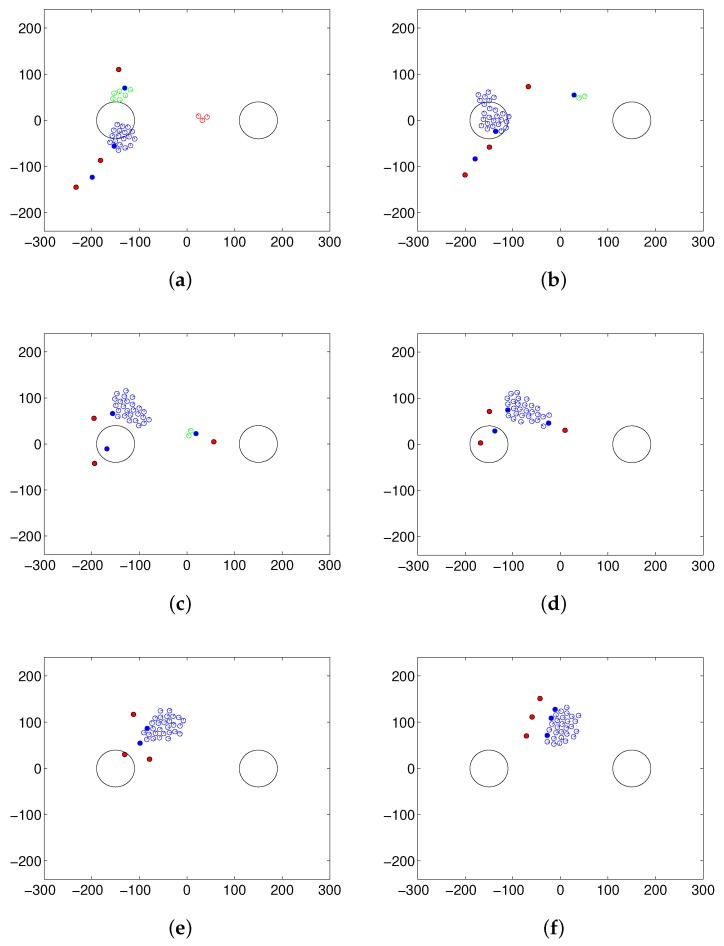
Snapshot of the simulation for performing collecting behavior over four scattered flocks: (**a**) patrolling and collecting, (**b**–**d**) herding and collecting and (**e**,**f**) herding after collecting all the scattered flocks.

Figure 6 shows an example of collecting four separated flocks into one large group with three steering robots and four separated flocks. In Figure 6a, there are more flocks than steering robots. One of the steering robots close to the largest flock (blue flock) performs the patrolling task, while the other steering robots collect the separated flocks until the number of flocks becomes the same as the number of steering robots. In Figure 6b–d, there is the same number of flocks as steering robots. The robot controlling the largest flock drives the flock to the desired location, while the other steering robots guide the other flocks toward the main flock. After collecting the scattered flocks, they are herding together to the desired area, as shown in Figure 6e,f.

### 5.3. Effect of the Number of Robots

**Figure 7:**
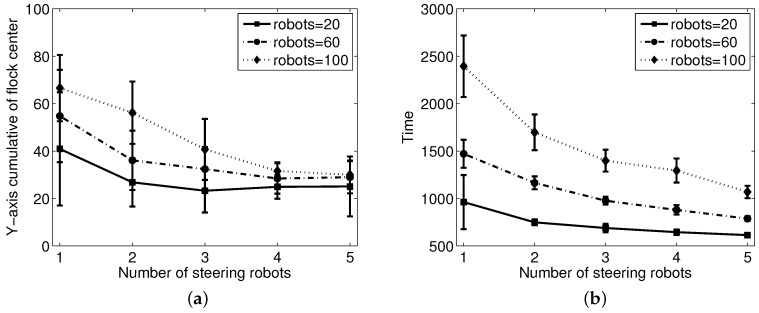
Comparison of varying numbers of steering and sheep robots: (**a**) cumulative score of the y-position deviation of the center of the sheep flock from the y-axis and (**b**) time needed to reach the goal area.

From the previous experiment, we see that paths vary according to the number of steering agents. Figure 7 shows how path accuracy, as assessed by the accumulated difference of the y-position value of the center of flock from the y-axis, and the time needed to reach the goal area depend on the number of steering agents and flock members. For each experiment, we repeated 10 independent runs and calculated the average performance in each case.

From the results, we can see that the time taken to arrive at the desired area decreases as the number of steering agents increases. In addition, as group size is changed from ten to one-hundred, more steering robots are needed to guide the group equally quickly. This is expected; however, if there are five steering-agents, performance does not change significantly versus utilizing fewer steering-agents. This means that for sufficiently large groups, only a very small number of informed individuals is needed to achieve the steering task. The interaction between a group of steering agents and a flock of sheep agents is controlled by the scaling parameters. Especially, $K_{t}$ is directly related to the performance of herding the flock to the goal area. With proper parameters, even one steering robot can control 100 sheep robots. However, a group of sheep agents themselves cannot arrive at the goal without control input $u_{g}$.

### 5.4. Effect of Speed of Robots

**Figure 8:**
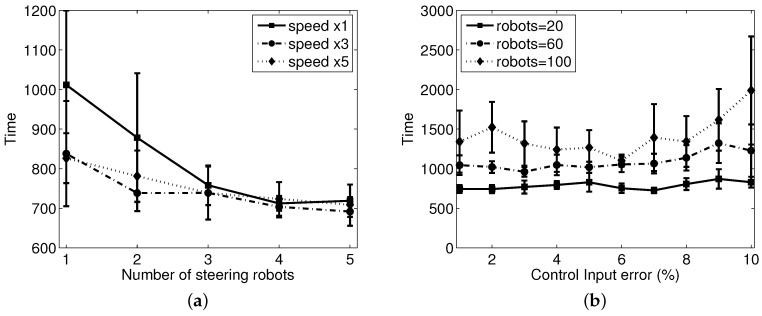
Results with the change in the speed of steering robots: (**a**) time to reach the goal area vs. the speed of the steering robot and (**b**) time vs. control input error.

To analyze the effect of the moving speed of robots, we change the moving speed of the steering agents while the speed of the sheep agents keeps constant with the previous simulation. Figure 8a shows the results when the moving speed of steering robot is three- and five-times faster than the sheep robots. There were 30 sheep robots in this experiment, and it shows that the time needed to reach the goal decreases as the number of steering agents increases. This result is consistent with the previous results. However, we note that the effect of the moving speed of the shepherding robot can be observed under limited conditions for which there are only one or two steering agents, and the speed is the same as the sheep agent. With multiple steering robots, the performance is less affected by the herding speed. Figure 8b shows the result when there are three steering agents and different levels of noise errors are added to the control input, $u_{i}$. The performance becomes worse slightly proportional to the input control error with a large flock of sheep agents. However, it seems that the overall performance is well maintained, and the current shepherding system is robustly working for a reasonable size of sheep agents.

### 5.5. Effect of Scaling Parameters

**Figure 9:**
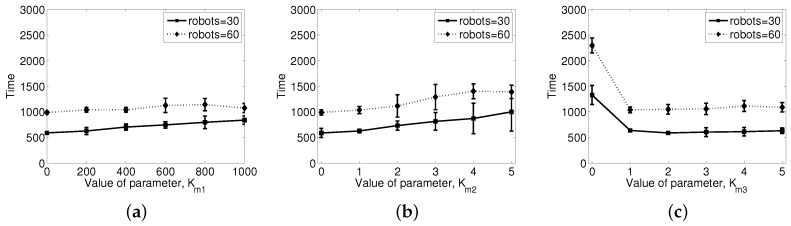
Time needed to reach the goal area, depending on changes in the parameters for forming a flock: (**a**) $K_{m1}$ for keeping distance from other sheep agents; (**b**) $K_{m2}$ for remaining close to the flock; and (**c**) $K_{m3}$ for velocity matching among sheep agents.

We investigate how the system responds to the set of scaling parameters mentioned above. The shepherding task was tested with three steering agents for 30 and 60 sheep agents. In the control algorithm, there are several variables: scaling parameters, the range of perception, the size of the flock and the parameters of the noise distributions. The scaling parameters are mainly classified into two categories, namely parameters to form a flock of sheep agents and parameters to perform the shepherding action for the steering agents. Figure 9 shows the effect of parameters on the formation of a flock in the shepherding task; $K_{m1}$ is related to the distance kept from other agents, $K_{m2}$ is used to remain close to the flock, and $K_{m3}$ is related to the velocity matching among sheep agents. From the results, we note that $K_{m1}$ and $K_{m2}$ have a negative effect on the herding performance, whereas $K_{m3}$ has a positive effect on the herding performance. Due to the cut-off distance in the gradient of the potential function, $K_{m1}$ and $K_{m2}$ have a limited effect. Nevertheless, the shepherding task is not possible if $K_{m3}$ is set to zero. This term is related to the velocity matching among the sheep agents, and in order to improve the herding performance, the movement speed of the steering agents is often transferred to the sheep agents. The shepherding task is based on the assumption that the flock is maintained well. We can set each parameter to form a flock, as well as to respond to the steering agents properly.

**Figure 10:**
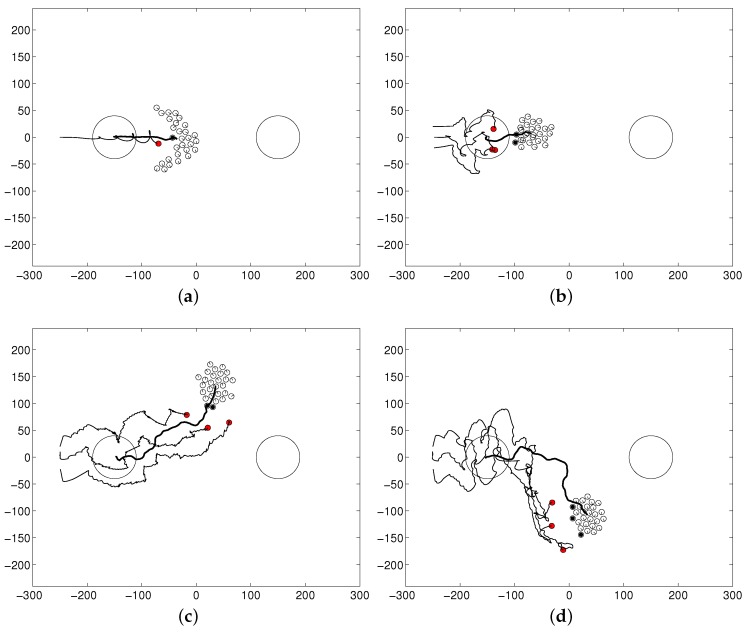
Examples of agent behaviors missing the goal: (**a**) the flock of sheep agents is scattered; (**b**) arc formation is not generated; (**c**) steering agents move in the wrong direction; and (**d**) the flock is not controlled by the steering agents.

Figure 10 shows various cases for which the flock of sheep agents is not controlled well to achieve the desired goal by changing the parameters related to the shepherding task, namely $K_{t}$ for the sheep agents and $K_{m}^{\prime }$, $K_{t}^{\prime }$ and $K_{g}^{\prime }$ for the steering agents. Figure 10a shows the result when $K_{t}^{\prime }$ is not large enough, for example when $K_{t}^{\prime }=10$. This parameter controls the collision avoidance of steering agents from the flock. If the steering agent moves too close to the center of the flock, it changes the shape of the flock. Therefore, we used a high value of 5000 for the parameter $K_{t}^{\prime }$ to prevent the flocking pattern from being scattered. If the flocking tendency among the sheep agents is not strong, the flock collapses easily into a scattered pattern. Figure 10b shows the result when $K_{m}^{\prime }$ is changed from the normal setting of 800 to 10. This parameter controls the interaction among the steering agents. It can be seen that collision avoidance and arc formation among the steering agents were not induced. Figure 10c,d shows the results when $K_{g}^{\prime }$ and $K_{t}$ are changed to 0.002 and 10, respectively from the normal setting of $K_{g}^{\prime }=1$ and $K_{t}=500$. It can be seen that by setting the parameters $K_{o}$ and $K_{t}$ with these small values, the steering agents drove the flock to the goal, and the sheep robots moved away from the approaching steering agents, respectively. However, since both terms are too small, either the steering agents moved in the wrong direction or the flock was not controlled well.

**Figure 11:**
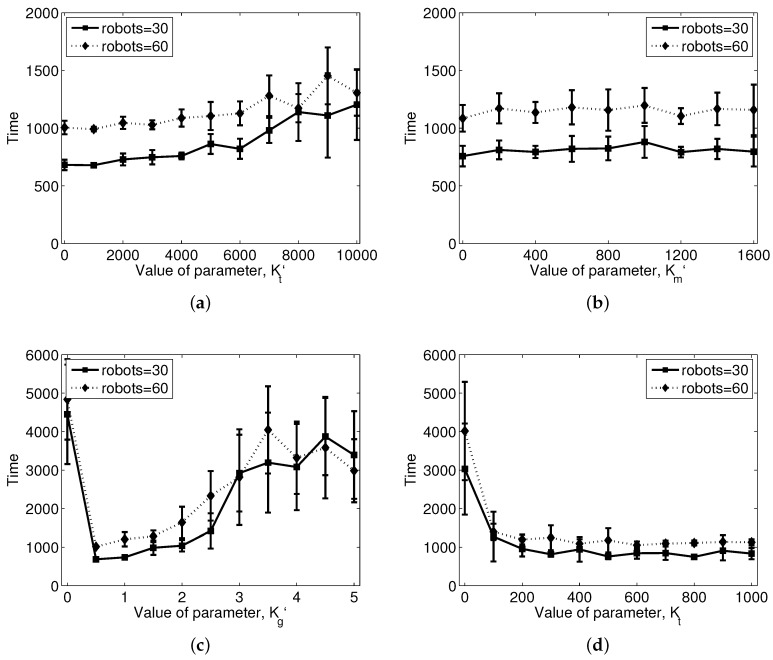
Time needed to reach the goal area with changes in parameters for the shepherding task: (**a**) $K_{t}^{\prime }$ controls the collision avoidance of the steering agents from the flock; (**b**) $K_{m}^{\prime }$ controls the collision avoidance among the steering agents; (**c**) $K_{g}^{\prime }$ drives the flock to the goal area; and (**d**) $K_{t}$ causes the sheep agents to move away from the steering agents; (**a**–**c**) are for the steering agents, and (**d**) is for the sheep agents.

A more detailed analysis of the parameters to achieve the shepherding task is shown in Figure 11. It shows the ranges of parameters that are valid for the shepherding task. We evaluated the amount of time required to complete the task, that is the time required for a flock of sheep agents to arrive at the destination area. If the flock does not reach the goal, the result is set to the maximum simulation time, which is 5000 time units. Figure 11a shows the result of the interaction between the steering agents and sheep agents by changing the parameter $K_{t}^{\prime }$. As the value of the parameter increases, time needed to complete the task increases. With high $K_{t}^{\prime }$, a set of steering agents moves slowly towards the sheep agents, which delays the shepherding task. In contrast, small $K_{t}^{\prime }$ has the potential to move directly towards the center of the flock, and this could scatter the flock into subgroups if there is weak cohesion among the sheep agents to form a flock. In Figure 11a, the flock is not scattered with small $K_{t}^{\prime }$, due to the strong cohesion among the sheep agents to form a flock and the collecting behavior of their steering agents. In this way, many parameters are intertwined together for the shepherding task. Figure 11b shows the result of the interaction among the steering agents when $K_{m}^{\prime }$ is changed. It seems that this parameter does not have any effect on decreasing the time required to reach the goal. However, it is definitely required for collision avoidance among the steering agents. In addition, if the flocking cohesion is weak, the arc formation among the steering agents will help with improving the performance by preventing the escape from the flock. Figure 11c shows the result of the change in $K_{g}^{\prime }$. This parameter is related to the map information, and the flock cannot reach the goal without this term. The performance decreases as the value increases to a high level, since too much concentration on the goal disturbs the movement of steering agents on the right track, ultimately influencing the movement path of the flock. Figure 11d shows the result of the change in $K_{t}$. The performance converges as the value increases. From the results, it can be seen that the overall performance is changed depending on several factors. An optimized setting of parameters can be searched to improve the performance; however, it is left as our future work.

The control term, $u_{g}$, in Equation (10) is an essential term for herding a flock to the desired destination, while the other terms are related to maintaining the flock or avoiding obstacles. We can say that a shepherding robot has a pushing force of moving the flock, since the sheep agents try to avoid the shepherding robot. If a steering robot is far away from the flock or the flock is away from the desired target location, the control term $u_{g}$ moves the steering robot to the opposite direction of the target. It gradually reduces the distance gap between the flock and the target position. If the flock is maintained well, the flock can be guided to the desired goal due to this control input. The interaction between the flocking behavior among the homogeneous group and the shepherd-avoiding behavior of the flock may influence the stability of the performance. However, if prior conditions for flocking of sheep agents are satisfied, their movement to the target can be completed without difficulty. The coordination of steering robots to move the sheep agents can be another factor that affects the performance. An inhibition among steering agents allows them to keep space among them and efficiently control the sheep agents. The appropriate choice of parameters can achieve the stability of convergence to reach the target position. In our experimental setup with control parameters, the flock moved to the destination without failure over many trials.

## 6. Conclusions

In this paper, we proposed a flocking control algorithm for multiple steering agents (i.e., shepherding behavior). Shepherding behavior is a flocking behavior, in which one or more external agents (steering agents) guide another group of sheep agents). In nature, we can observe that many animals moving in groups decide their movement depending on interactions among group members. Only a few individuals in groups have unique information, such as knowledge of the location of a food source or a migration route from the current area to the desired area. This behavior is easily found in nature, that is in shepherding, the sheep react to the shepherding animal by moving away from it. These kinds of problems have been studied in various disciplines, from biology to robotics. There is evidence that individuals within groups have access to local information about the behavior of near-neighbors when moving around obstacles or avoiding predators [49].

In this paper, we designed control rules to implement shepherding behaviors with multiple steering robots without centralized coordination. The task was to move the flock to the desired location. We assume that the steering robot can be monitored by a camera, and its current position relative to the goal area is measured. If the information of the goal area is not available, the shepherding algorithm may not work. At least the direction towards the goal should be given to achieve the task. In our proposed method, a set of steering agents only needs information from locally observing sheep agents, not all members of the group. The steering agents only focus on guiding the nearest sheep agent to the desired location without considering any other agents in the flock. Yet, some sheep agents detect their approach and try to move away from the steering agents; this tendency produces a group behavior of the flock to move towards the destination. The interactions among steering agents are also controlled autonomously, not manually. In addition, even if some sheep agents are far away and are not a part of the primary flock, they can be merged into the flock and guided home, by the collecting behavior of shepherding agents. This method can be applied to real shepherding work or other similar systems. In most systems, localization is carried out by a centralized system that uses information provided by all agents. However, in the real world, that type of centralized system is often infeasible. For example, observing all the sheep cannot be achieved by one shepherd dog. Thus, our approach is more applicable to real-world situations.

The flocking tendency among sheep agents and the steering ability of shepherding agents are affected by several factors, that is a set of control parameters given in Table 1, and thus, the performance can be changed. If the flock is maintained and controlled well under the proper conditions, the flock can be guided to the desired goal. The collecting behavior of steering agents helps with merging sheep agents into a flock even when the flock is scattered. With the flocking tendency of sheep agents, a goal-directed factor derived by the vector $u_{g}$ in Equation (10) can herd the flock into the target zone, and thus, the flock follows a path to the target. In this paper, we did not prove the convergence stability of the flock moving to the target. It was shown that the appropriate choice of control parameters can successfully guide the flock. Its theoretical foundation or proof is left as a future work.

By assessing the arrival time and path accuracy of the flock in reaching the desired position, we can observe the effect of the suggested control algorithm. From the simulation experiment, we showed that steering control of the swarm was possible without centralized coordination, and this control could successfully adapt to different mission conditions, such as patrolling, covering and collecting. Only a small number of steering agents are needed, and they can trace arc paths autonomously to control the flock effectively. Even scattered flocks can be merged into one flock. In the future, we aim to develop more optimized methods for the shepherding task, by adapting more intelligent algorithms. Many factors are involved with the shepherding task in our model, and the effect of changing the value of individual parameters on the behavior of sheep and shepherds is analyzed. We will search for optimized parameters to improve the performance, using evolutionary computation. In addition, we wish to test various steering tasks in reality by building a swarm of real mobile robots.

## Figures and Tables

**Table 1:** Description of the parameters in the control algorithm: (**a**) list of all parameters; (**b**) behavior tendency corresponding to the scaling parameters.

(**a**)	
**Description**	**Symbol**
coordination parameters	$x_{i}$, $y_{i}$, $p_{i}$, $p_{j}$, $q_{i}$, $q_{j}$, $q_{t}$, $q_{g}$
control inputs	$u_{i}$, $u_{m}$, $u_{m1}$, $u_{m2}$, $u_{m3}$, $u_{o}$, $u_{t}$, $u_{g}$
scaling parameters	$K_{m1}$, $K_{m2}$, $K_{m3}$, $K_{o}$, $K_{t}$, $K_{m}^{\prime }$, $K_{o}^{\prime }$, $K_{t}^{\prime }$, $K_{g}^{\prime }$
sets of agents or obstacles	$N_{m}$, $N_{o}$, $N_{t}$
(**b**)	
**Symbol**	**Behavior Tendency**
$K_{m1}$	Keep distance from other sheep agents
$K_{m2}$	Remain close to the neighboring sheep agents
$K_{m3}$	Velocity matching among neighboring sheep agents
$K_{t}$	Collision avoidance of sheep agent from steering agents
$K_{t}^{\prime }$	Collision avoidance of steering agent from sheep agents
$K_{m}^{\prime }$	Keep distance from other steering agents
$K_{g}^{\prime }$	Herd the flock to the goal area
$K_{o}$	Collision avoidance of sheep agent from obstacles
$K_{o}^{\prime }$	Collision avoidance of steering agent from obstacles
